# Supplementary material for: Probabilistic nested model selection in pharmacokinetic analysis of DCE-MRI data in animal model of cerebral tumor
Source: Sci Rep. 2025 Jan 13;15:1786. doi: 10.1038/s41598-024-83306-6 (PMC11729890; doi:10.1038/s41598-024-83306-6)
Supplement: Supplementary file 1 — Supplementary Material 1 [file 41598_2024_83306_MOESM1_ESM.docx]

**Abbreviations:**

Dynamic Contrast Enhanced (DCE)

Dual Gradient Echo (DGE)

Nested Model Selection (NMS)

Probabilistic Nested Model Selection (PNMS)

Kohonen Self Organizing Map (K-SOM)

Dice Similarity Coefficients (DSCs)

Nested Cross Validation (NCV)

Random Permutation Sampling (RPS)

Contrast Agent (CA)

The longitudinal-relaxivity change (ΔR_1_)

Confidence Level (CL)

Confidence Interval (CI)

Pharmacokinetic (PK)

Best Matching Unit (BMU)

T One by Multiple Read Out Pulses (TOMROP)

Institutional Animal Care and Use Committee (IACUC)

Mean Percent Difference (MPD)

Lower Bound (LB)

Upper Bound (UB)

Akaike’s Information Criteria (AIC)

Akaike’s Information Criteria corrected (AICc)

Bayesian information criteria (BIC)

The ARRIVE guidelines (Animal Research: Reporting of In Vivo Experiments)
